# Microfluidic Platforms Promote Polarization of Human-Derived Retinal Ganglion Cells That Model Axonopathy

**DOI:** 10.1167/tvst.12.4.1

**Published:** 2023-04-03

**Authors:** Andrew M. Boal, Nolan R. McGrady, Xitiz Chamling, Bhanu S. Kagitapalli, Donald J. Zack, David J. Calkins, Michael L. Risner

**Affiliations:** 1 Vanderbilt Eye Institute, Department of Ophthalmology & Visual Sciences, Vanderbilt University Medical Center, Nashville, TN, USA; 2 Wilmer Eye Institute, Department of Ophthalmology, Johns Hopkins University School of Medicine, Baltimore, MD, USA

**Keywords:** human embryonic stem cells, retinal ganglion cells, axon initial segment, axonopathy, glaucoma

## Abstract

**Purpose:**

Axons depend on long-range transport of proteins and organelles which increases susceptibility to metabolic stress in disease. The axon initial segment (AIS) is particularly vulnerable due to the high bioenergetic demand of action potential generation. Here, we prepared retinal ganglion cells derived from human embryonic stem cells (hRGCs) to probe how axonal stress alters AIS morphology.

**Methods:**

hRGCs were cultured on coverslips or microfluidic platforms. We assayed AIS specification and morphology by immunolabeling against ankyrin G (ankG), an axon-specific protein, and postsynaptic density 95 (PSD-95), a dendrite-specific protein. Using microfluidic platforms that enable fluidic isolation, we added colchicine to the axon compartment to lesion axons. We verified axonopathy by measuring the anterograde axon transport of cholera toxin subunit B and immunolabeling against cleaved caspase 3 (CC3) and phosphorylated neurofilament H (SMI-34). We determined the influence of axon injury on AIS morphology by immunolabeling samples against ankG and measuring AIS distance from soma and length.

**Results:**

Based on measurements of ankG and PSD-95 immunolabeling, microfluidic platforms promote the formation and separation of distinct somatic–dendritic versus axonal compartments in hRGCs compared to coverslip cultures. Chemical lesioning of axons by colchicine reduced hRGC anterograde axon transport, increased varicosity density, and enhanced expression of CC3 and SMI-34. Interestingly, we found that colchicine selectively affected hRGCs with axon-carrying dendrites by reducing AIS distance from somas and increasing length, thus suggesting reduced capacity to maintain excitability.

**Conclusions:**

Thus, microfluidic platforms promote polarized hRGCs that enable modeling of axonopathy.

**Translational Relevance:**

Microfluidic platforms may be used to assay compartmentalized degeneration that occurs during glaucoma.

## Introduction

Human pluripotent stem cells differentiated into specialized cells of the central nervous system are used to model several neurodegenerative diseases, including glaucoma.^1^^–^^6^ Glaucoma is an age-related neurodegenerative disease exacerbated by increased sensitivity of visual tissues to translaminar pressure gradients at the optic nerve head, a transition zone where unmyelinated retinal ganglion cell (RGC) axons project through the optic nerve.^7^ Stress transduced at the optic nerve head piques distal nerve fibers, activating mechanisms that promote retrograde axonopathy.^8^^,^^9^

Previously, we generated model RGCs from human embryonic stem cells (hESCs) using CRISPR Cas9 to express tdTomato and THY1.2 under the control of *BRN3B*, a gene enriched in native RGCs. We differentiated BRN3B-tdTomato-THY1.2 hESCs toward a RGC fate by chemical induction and immunopurified cells by targeting the surface protein THY1.2.^6^ During differentiation, hESC-derived RGCs (hRGCs) recapitulated developmental milestones similar to endogenous RGCs, including increased expression of *ATOH7*, *BRN3B*, *ISL1*, and *SOX4* transcripts and RNA-binding protein with multiple splicing (RBPMS) and TUJ1 proteins.^6^^,^^10^

Recently, we investigated morphologic and physiologic differentiation of hRGCs in vitro. We confirmed that hRGCs are highly pure, with 98.5% of tdTomato-positive cells co-expressing RBPMS.^11^^,^^12^ In vitro, hRGC neurites continually grew up to at least 4 weeks, and, as neuritic fields expanded, postsynaptic densities localized to neurites. In addition to dendrite-specific proteins, hRGCs expressed genes encoding axon-related proteins, including ankyrin G (ankG),^12^ which is a scaffolding protein that organizes constituent proteins composing the axon initial segment (AIS). The several functions that ankG serves include maintaining neuron polarity, recruitment of voltage-gated channels essential for electrogenesis, and scaling AIS geometry to adapt to changes in excitability.^13^^–^^18^ The expression of ankG is targeted by axonopathies, yet ankG is required for regeneration and reinnervation following axon injury.^19^^,^^20^ Therefore, ankG is a prime indicator of neuronal differentiation, degeneration, and targets for repair.

Although we previously found that hRGCs expressed the gene (*ANK3*) encoding ankG, hRGCs plated on coverslips without supplementation with growth factors weakly expressed ankG protein early during differentiation, and ankG appeared irregularly localized during later time points. In agreement with other reports in human pluripotent stem RGCs,^21^^,^^22^ we found that hRGC current-clamp responses were sensitive to depolarization block, indicative of immature neurons.^12^^,^^23^ Based on protein and physiologic measurements, hRGCs cultured on coverslips without supplementation with growth factors appear not to polarize robustly intrinsically.

Here, we first demonstrated that hRGC axon specification is enhanced when hRGCs are cultured on microfluidic platforms compared to coverslips.^24^^–^^26^ Based on ankG immunolabeling, hRGCs plated on coverslips and microfluidic platforms possessed three distinct AIS localizations: ankG localized to a neurite directly stemming from the soma (direct), ankG accumulated on an axon-carrying dendrite (AcD),^27^^,^^28^ or ankG enriched within multiple processes (multi). Notably, 25% of cells plated on coverslips contained multiple ankG-labeled neurites, indicating a lack of axon specification. Only 5% of cells possessed multiple AISs in microfluidic platforms. We then quantified AIS length and distance from the soma, as these dimensional variables correlate with voltage-gated channel conductance^29^^,^^30^ and are altered in models of degeneration.^30^^–^^32^ We found that microfluidic platforms normalized hRGC AIS length and distance compared to coverslip cultures relative to AIS morphologies of mouse RGCs from whole-mount retinas.

We then leveraged our hRGC microfluidic culture system to model axonopathy induced by colchicine, which has been previously used to promote RGC degeneration.^5^^,^^6^^,^^33^ After three days of colchicine treatment in the axon chamber, we found hRGC axons degraded, as indicated by increased varicosities, loss of anterograde transport of cholera toxin subunit B, axon retraction, and outright degeneration. Moreover, we found evidence that hRGCs with AcDs are sensitive to colchicine treatment compared to direct AISs. For cells with AcDs, colchicine reduced AIS distance from the soma, suggesting diminished excitability.^34^ Overall, our findings indicate that compartmentalized microenvironments promote polarization of hESC-derived neurons, thus enabling in vitro modeling of axonopathies, such as glaucoma.

## Methods

### Animals

All experimental procedures were approved by the Institutional Animal Care and Use Committee of Vanderbilt University Medical Center and aligned with the ARVO Statement for the Use of Animals in Ophthalmic and Vision Research. B6.Cg-Tg(Thy1-YFP)16Jrs/J (strain #003709) mice were purchased from The Jackson Laboratory (Bar Harbor, ME). Upon delivery, the mice were housed at the Division of Animal Care facilities at Vanderbilt and provided water and standard chow ad libitum. The strain was maintained by breeding homozygotes with wild-type C57Bl/6J. For this study, we used heterozygous 5-month-old female mice. We used female mice for comparison because H9 hESCs are of female origin.

### Cell Culture

We obtained H9 BRN3B-P2A-tdTomato-P2A-THY1.2 hESCs differentiated by chemical induction for 40 days toward a RGC fate from Donald Zack, MD, PhD.^6^^,^^12^ hRGCs were shipped frozen overnight from The Johns Hopkins University to Vanderbilt University Medical Center; upon arrival, we immediately stored the hRGCs in liquid nitrogen. The hRGCs were then thawed and suspended in culture medium. Culture medium consisted of BrainPhys (#05790; STEMCELL Technologies, Vancouver, BC, Canada), 1% N2 Supplement-A (#07152; STEMCELL Technologies), 2% NeuroCult SMI1 Neuronal Supplement (#05711; STEMCELL Technologies), 20-ng/mL human recombinant brain-derived neurotrophic factor (BDNF, #78005; STEMCELL Technologies), 20 ng/mL human recombinant glia-derived neurotrophic factor (GDNF, #78058; STEMCELL Technologies), 200-nM ascorbic acid (#72132; STEMCELL Technologies), 1-mM Adenosine 3′,5′-Cyclic Monophosphate N^6^,O^2^^′^-Dibutyryrl-, Sodium Salt (#28745; Sigma-Aldrich, St. Louis, MO), and 1% Gibco gentamicin (#15750060; Thermo Fisher Scientific, Waltham, MA). We then centrifuged the cell suspension at 120*g* for 5 minutes. Following centrifugation, we removed the supernatant, which included dimethyl sulfoxide–based cryopreservation medium (#07930; STEMCELL Technologies), and resuspended the cell pellet in fresh culture medium.

We plated hRGCs on 18-mm glass coverslips (#72222-01; Electron Microscopy Sciences, Hatfield, PA) contained in 12-well plates (#665180; Greiner Bio-One, Kremsmünster, Austria) or XC450 microfluidic platforms (Xona Microfluidics, Triangle Park, NC). Coverslips and microfluidics were coated with 50-µg/mL poly-d-lysine (#354210; Corning Inc., Corning, NY) diluted in sterile Dulbecco's phosphate-buffered saline (DPBS) overnight at 37°C followed by incubating culture platforms in 10-µg/mL mouse laminin (#23017015; Thermo Fisher Scientific) diluted in DPBS for 4 hours at 37°C. For microfluidic platforms, we added 75,000 cells per soma access port. However, only about 75% of these cells were drawn into the soma chamber. We plated hRGCs on coverslips at a density of 60,000/cm^2^. Samples were incubated at 37°C with 5% CO_2_. The day after plating cells, we exchanged half of the medium in each port with fresh culture medium, adding an additional 30 ng/mL BDNF to the coverslip cultures and to the axon chambers of the microfluidic platforms. For microfluidic cultures, we maintained a volume difference between the soma (160 µL) and axon chambers (120 µL) to drive neurite outgrowth toward the axonal chamber. Subsequently, we exchanged half of the medium every 2 to 3 days for the duration. Cultures were maintained for 10 to 15 days. Following each endpoint, we fixed samples in 4% paraformaldehyde (PFA).

For a subset of experiments, we induced degeneration of putative axons by administering colchicine (#C9754; Sigma-Aldrich), an agent that inhibits microtubule polymerization. Prior to adding the colchicine, we verified that axons projected into the axon chamber by imaging live samples using epifluorescence microscopy. After 8 to 10 days in vitro (DIV), we typically observed axons filling the microgroove section and emerging into the axon chamber. After confirming axons crossing into the axon chamber, we cultured cells an additional 48 hours to allow for further axon elongation before treating cells with colchicine or vehicle. After 10 to 12 DIV, we added 30-nM colchicine into the access ports serving the axon chamber. Colchicine stocks were dissolved in sterile DPBS and further diluted with culture medium for working solutions. For vehicle samples, we added fresh culture medium to the ports serving the axon chamber. Samples were incubated in colchicine for 3 days.^5^ Twelve hours prior to the endpoint, we added 1% cholera toxin subunit B (CTB, #C34778; Thermo Fisher Scientific) diluted in culture medium into the soma chamber access ports to track anterograde axonal transport. Afterward, we fixed samples for 30 minutes in 4% PFA diluted in phosphate-buffered saline (PBS) with azide.

### Immunocytochemistry and Imaging

Following fixation, we washed samples three times with PBS with azide and blocked with 0.1% Triton X-100 and 5% normal donkey serum (NDS) at room temperature for 2 hours. After blocking, we incubated samples overnight at 4°C in primary antibodies including postsynaptic density 95 (PSD-95, 1:300, #51-6900; Thermo Fisher Scientific), ankG (1:200, #75-146; NeuroMab, Davis, CA), cleaved caspase 3 (CC3, 1:400, #9661; Cell Signaling Technology, Danvers, MA), phosphorylated neurofilament H (SMI-34, 1:1000, #835501; BioLegend, San Diego, CA) in addition to 0.1% Triton X-100 and 5% NDS. The following day, we washed samples three times with PBS and incubated samples in 0.1% Triton X-100, 5% NDS, and appropriate secondary antibodies (Jackson ImmunoResearch, West Grove, PA) for 2 hours at room temperature. Afterward, samples were washed three times with PBS, and we applied Fluoromount-G (#0100-20; SouthernBiotech, Birmingham, AL). We imaged samples at the Vanderbilt University Nikon Center for Excellence. We used a Nikon (Tokyo, Japan) spinning disk confocal microscope with 40× or 60× oil-immersion objectives (Plan Apochromat Lambda, NA 1.40, WD 0.13 mm) equipped with a CSU-X1 spinning disk head (Yokogawa, Tokyo, Japan), Prime 95B sCMOS camera (Teledyne Photometrics, Tucson, AZ), automated stage driver, and four laser lines (405, 488, 561, and 647 nm). Exposure and laser power settings were kept constant across independent variables (tissue/cell culture preparation) for each dependent variable (e.g., ankG secondary antibody).

### Image Analysis

Immunofluorescence and colocalization were determined using ImageJ plugins (National Institutes of Health, Bethesda, MD). For AIS bounds determination, hRGC neurites containing ankG labeling were hand traced in ImageJ using the freehand line tool from the edge of the soma and past the bounds of visible ankG labeling. For each trace, the fluorescence profile from the ankG channel was exported, and the AIS localization (i.e., direct, AcD, or multi) was noted. The ankG profiles were analyzed in Python 3.9 using the SciPy 1.7.1 module.^35^ Background fluorescence was subtracted from the ankG intensity profiles using a rolling ball filter with a radius of 50. Smoothed ankG profiles were generated using a Savitzky–Golay filter with a first-order polynomial fit. AIS bounds were systematically defined as the extent where smoothed ankG values were greater than 50% of the difference between baseline and maximum intensity. Colocalization analysis was performed using the Coloc 2 plugin. For representative images, we enhanced immunofluorescence by subtracting background intensity, smoothing the intensity values, and increasing the contrast of the image.

### Statistical Analyses

All data are reported as mean ± SE of the mean. All statistical tests were performed in Prism 9 (GraphPad Software, San Diego, CA). All datasets were checked for normality. Two outliers for PSD-95 labeling data, one from each chamber as identified by Grubb's test with an alpha of 0.05, were excluded. There were a few instances (0% of mouse, 6% of coverslip, and 1.5% of microfluidic cultures) where AIS bounds could not be determined algorithmically due to weak immunofluorescence; these values were included for localization analysis but excluded for morphologic analyses. Cells with multiple AISs were also excluded from dimension analysis because a primary axon could not be determined. For colchicine-treated microfluidic devices, two of six devices were excluded from dimension analysis because they contained too few identifiable AISs of each localization type. Otherwise, all data were included in the analyses. For statistical tests on PSD-95 labeling, AIS dimension analysis, and colchicine degeneration assays, we used the average value from each independent replicate/sample for statistical tests. We defined statistical significance as *P* ≤ 0.05. Exact *P* values and sample sizes are indicated in the figure legends, alongside the specific details of the statistical test used for each analysis.

## Results

### Microfluidic Platforms Promote Normalization of hRGC Axon Initial Segment Morphology

**Figure 1.**
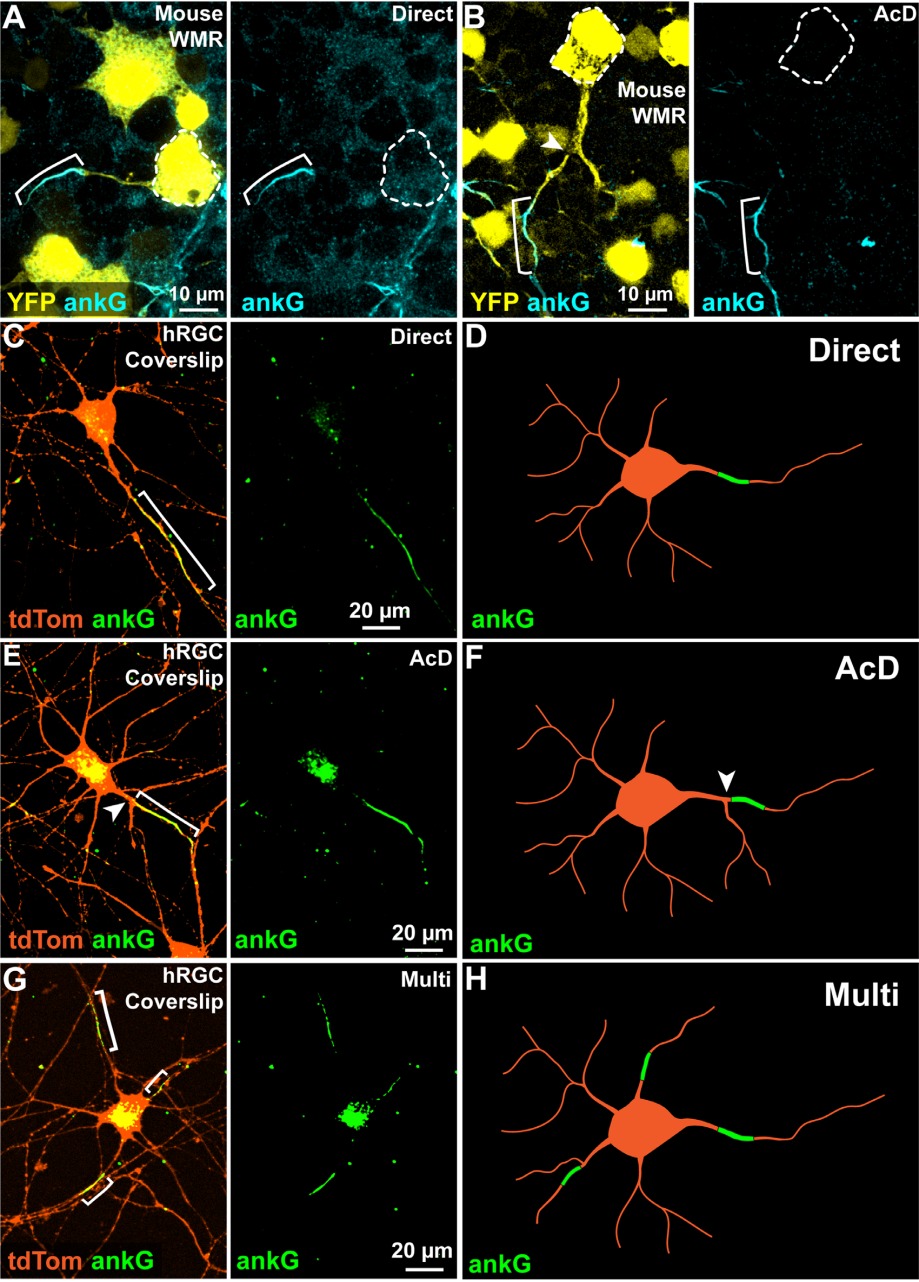
Mouse- and human-derived RGCs exhibit heterogeneity in AIS localization. (**A, B**) Representative micrographs of whole-mount retinas (WMRs) from B6.Cg-Tg(Thy1-YFP)16Jrs/J mice labeled for ankG demonstrate two distinct patterns of RGC AIS localization. (**A**) The majority of RGCs had a single ankG-labeled AIS (*cyan*) located on a process emanating directly from the YFP-labeled soma (*yellow*, with *white dotted outline*). (**B**) A small subset of mouse RGCs had an AIS located on a process distal to a bifurcation in a primary dendrite (*white arrowhead*), defined as an AcD. (**C–H**) Example micrographs of tdTomato (tdTom, *orange*) expressing RGCs derived from hRGCs cultured on coverslips demonstrate greater heterogeneity in ankG-labeled (*green*) AIS localization. As seen in mouse retina, hRGCs had a single AIS localized on a process emanating directly from the soma (direct, **C**; diagrammed in **D**) as well as on an AcD (**E**; diagrammed in **F**). There was also a subset of hRGCs that contained multiple AISs (multi, **G**; diagrammed in **H**).

We established a foundation for investigating AIS morphology in hRGCs by first demonstrating mouse RGC AIS localization. We immunolabeled whole-mount retinas from Thy1-YFP mice (female, 5 months of age, *n* = 3) against ankG. For the majority of mouse RGCs, we observed ankG localized to a single process emanating directly from a yellow fluorescent protein (YFP)-positive cell body (Fig. 1A). For a few mouse RGCs, ankG accumulated on a process distal to a bifurcation in a primary dendrite (Fig. 1B). This previously described AIS localization is referred to as an AcD.^27^^,^^28^^,^^36^

Next, we defined AIS localizations in hRGCs plated onto coverslips supplemented with BDNF and GDNF and cultured for 10 to 15 days. We observed three distinct AIS localizations defined by ankG immunolabeling. Similar to mouse RGCs, many hRGCs accumulated ankG within a single neurite extending directly from the soma (Figs. 1C, 1D) or enriched in an AcD (Figs. 1E, 1F). However, in addition to these two profiles, we also observed many instances of ankG localized to multiple neurites originating from a single soma (Figs. 1G, 1H).

**Figure 2.**
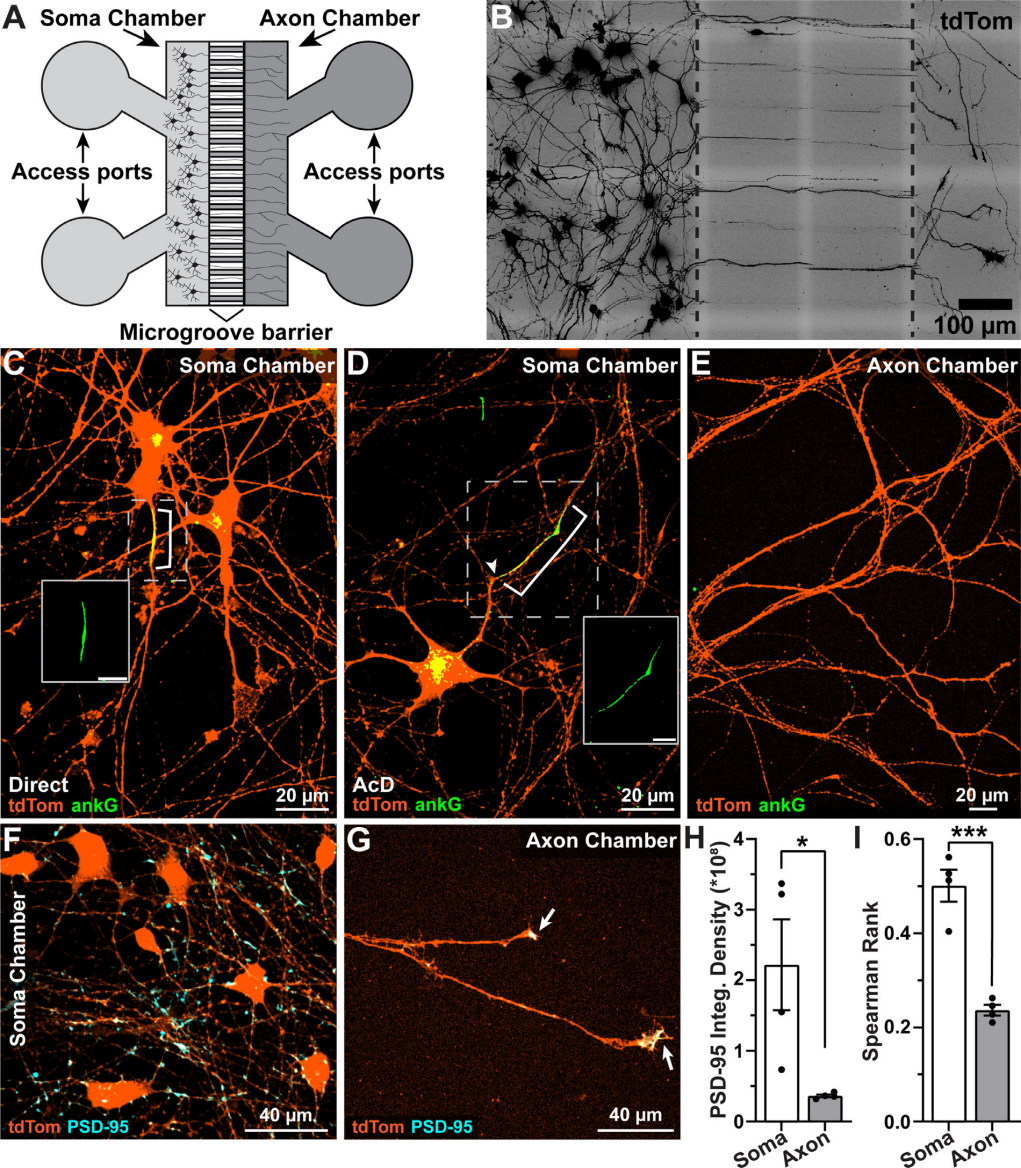
hRGCs plated on microfluidic platforms are polarized into somato–dendritic and axonal compartments. (**A**) Schematic of Xona XC450 microfluidic platform (not illustrated to scale). (**B**) After plating on the XC450 for 8 to 10 days, hRGC projections extended through the microgroove chamber and into the axon chamber. (**C**) When hRGCs were cultured in microfluidic platforms, ankG (*green*) typically localized to an axon extending directly from the soma (direct) or on an AcD within the soma chamber (**D**). Insets in C and D show ankG channel only. *Scale bars*: 10 µm. (**E**) ankG did not accumulate within putative axons in the axon chamber. (**F, G**) Representative micrographs of PSD-95–labeled (*cyan*) hRGCs cultured in microfluidic devices demonstrate evidence of postsynaptic specification in the soma chamber (**F**) and localization to putative axon growth cones in the axon chamber (**G**). *Arrows* indicate PSD-95 colocalized with tdTom-positive putative axon growth cones. (**H**) Quantification of PSD-95 immunofluorescence suggests significantly greater expression in the soma chamber than in the axon chamber (*P* = 0.0281, unpaired *t*-test). Integrated (Integ) density is the summed pixel values multiplied by area. (**I**) The soma chamber exhibited significantly greater colocalization of PSD-95 labeling and tdTom expression than did the axon chamber (*P* = 0.0003, unpaired *t*-test) (*n* = 4 independent devices). *Error bars:* ±SEM. *P < 0.05, ***P < 0.001.

We then determined the effect of culturing hRGCs in microfluidic platforms on AIS localization and polarization. hRGCs were plated onto microfluidic platforms supplemented with BDNF and GDNF and cultured for 10 to 15 days. Microfluidic platforms consisted of two primary chambers, soma and axon, connected by a microgroove barrier (Fig. 2A). After 8 to 10 DIV, we observed neurites extending through the microgroove section and into the axon chamber (Fig. 2B). After performing immunocytochemistry and confocal microscopy, we found that hRGCs cultured in microfluidic devices exhibited two principal AIS localizations: direct and AcD (Figs. 2C, 2D). Importantly, we did not observe ankG accumulation within putative axons projecting into the axon chamber (Fig. 2E), indicating restriction of ankG localization near the somas and not in distal axons.^13^ To further investigate hRGC polarization, we immunolabeled cells against the excitatory postsynaptic marker PSD-95 and determined PSD-95 integrated density and colocalization with tdTomato in the soma (Fig. 2F) and axon chambers (Fig. 2G). PSD-95 immunolabeling was significantly stronger, accounting for area, in the soma chamber (*P* = 0.0281) (Fig. 2H), and there was greater colocalization of PSD-95 labeling with tdTomato-positive hRGC processes in the soma chamber than in the axon chamber (*P* = 0.0003) (Fig. 2I). Although PSD-95 did not heavily accumulate within fibers projecting into the axon chamber, similar to other reports we observed PSD-95 localized within distal axon growth cones (Fig. 2G).^37^^,^^38^

**Figure 3.**
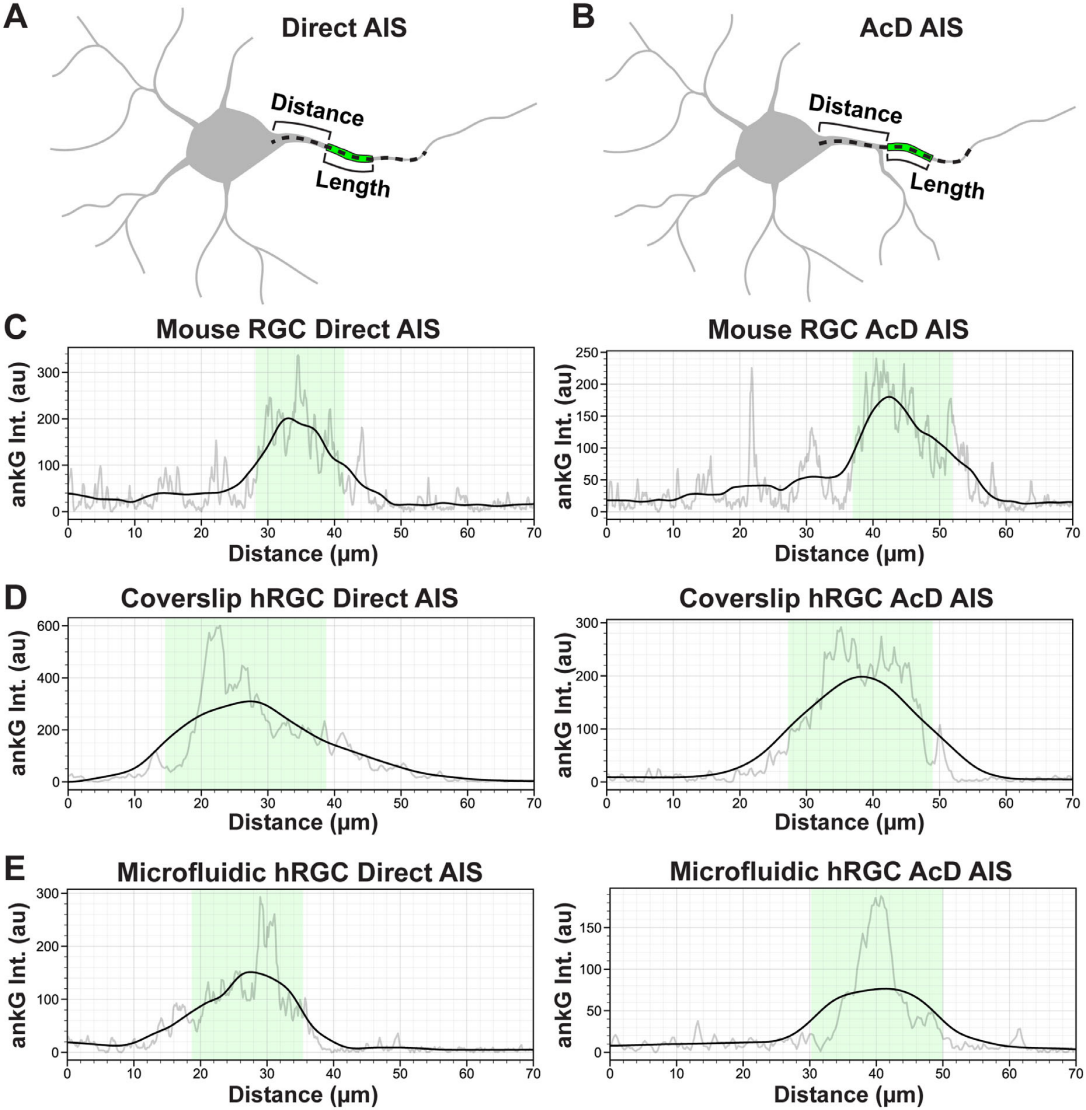
Systematic quantification of AIS length and distance from soma. (**A, B**) To determine AIS distance from soma and length, intensity profiles of ankG immunofluorescence were determined from hand traces (shown as *dashed line*) extending from the soma edge along the ankG-containing process for direct (**A**) and AcD (**B**) morphologies. The ankG is illustrated as a *green span* along the process. (**C–E**) Representative ankG intensity profiles from mouse RGCs (**C**), coverslip hRGCs (**D**), and hRGCs plated on microfluidic platforms (**E**). From raw traces (*light gray lines*), background fluorescence was subtracted, ankG intensity profiles were smoothed (*black lines*), and we defined the AIS length as the extent where the smoothed ankG intensity was greater than 50% of the difference between baseline and maximum intensity (*green shaded region*). au, arbitrary units; Int, mean intensity.

**Figure 4.**
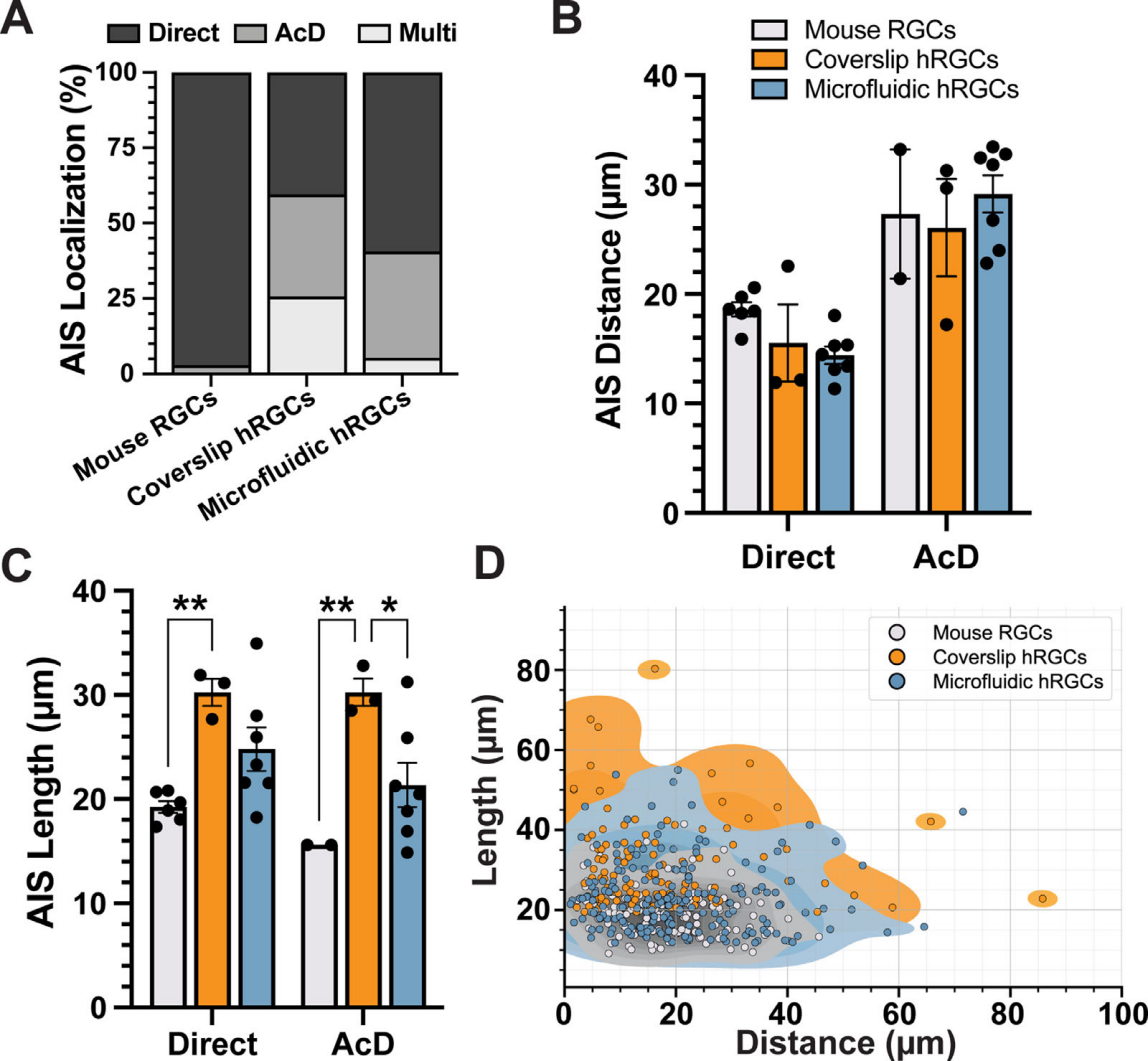
Microfluidic platforms promote normalization of AIS morphology in hRGCs. (**A**) Cumulative percentages of AIS localizations observed in mouse RGCs, hRGCs plated on coverslips, and hRGCs cultured in microfluidic platforms. (**B**) The AIS distance from the soma is significantly longer for AcD than direct AISs (*P* < 0.0001), but largely the same across cell types/culture platforms (*P* = 0.6999). (**C**) hRGCs cultured on coverslips possessed direct (*P* = 0.0041) and AcD (*P* = 0.0031) AISs significantly longer than mouse RGC direct and AcD AISs, respectively. The hRGCs plated on microfluidic platforms contained AcD AISs significantly shorter than hRGCs cultured on coverslips (*P* = 0.0182). The hRGCs plated on microfluidics possessed AISs of similar length to mouse RGCs (*P* ≥ 0.083). (**D**) Distribution of AIS distance versus length scatterplots for each of the cell types/culture platforms as determined by kernel density estimates. Coverslip hRGC (*orange*) AIS dimensions appear to have a more variable distribution than microfluidic hRGCs (*blue*) or mouse RGCs (*gray*). Sample sizes (excluding multi): mouse RGC group, 135 cells, six retinas (only two retinas contained AcD); coverslip hRGC group, 109 cells from three independent samples; microfluidic hRGC group, 190 cells, seven independent devices. Statistics: two-way ANOVA, Tukey post hoc test (**B, C**); kernel density estimate (**D**). *Error bars:* ±SEM. *P < 0.05, **P < 0.01.

We then established a systematic method for measuring AIS dimensions by tracing ankG fluorescence profiles along neurites and computationally determining AIS bounds (Fig. 3). We used this method to compare AIS dimensions among ankG localizations across RGCs from whole-mount retinas of mice, hRGC coverslip cultures, and hRGC microfluidic platform cultures (Fig. 4). For mouse RGCs, we found that 97% (131/135) of cells possessed an AIS stemming directly from the soma, and the remaining 3% (4/135) localized to an AcD (Fig. 4A). Of the hRGCs plated on coverslips with an AIS that could be directly traced back to its soma of origin, 40% (63/156) stemmed directly from the soma, 34% (53/156) localized to an AcD, and 26% (40/156) possessed multiple AISs (Fig. 4A). For hRGCs cultured on microfluidic platforms, we found 59.3% (121/204) of AISs localized to an axon projecting directly from the soma, 35.3% (72/204) stemmed from an AcD, and the remaining 5.4% (11/204) contained multiple AISs (Fig. 4A).

We then compared AIS dimensions for all cells possessing a single ankG-positive neurite (i.e., direct or AcD localizations). The distance from the edge of the soma to the beginning of the ankG labeling was significantly longer for AcD AISs than direct (*P* < 0.0001) (Fig. 4B). However, within each AIS type, AIS distance was similar among mouse RGCs and hRGC culture conditions (*P* = 0.6999) (Fig. 4B). Compared to mouse RGCs, hRGCs plated on coverslips possessed significantly longer direct and AcD AISs (direct *P* = 0.0041; AcD *P* = 0.0031) (Fig. 4C). hRGCs plated onto microfluidic platforms possessed significantly shorter AcD AISs versus hRGCs plated onto coverslips (*P* = 0.0182). Moreover, we found that microfluidic hRGC AIS lengths were similar to mouse RGCs for both direct (*P* = 0.0830) and AcD (*P* = 0.2861) AISs (Fig. 4C). Finally, we noted differences in the variability in AIS dimensions among mouse RGCs, hRGCs plated on coverslips, and hRGCs plated on microfluidic platforms. The hRGCs plated on coverslips appeared to have a highly variable distribution of the AIS distance versus length relationship, whereas microfluidic hRGC and mouse RGC AIS dimensions demonstrated less variability (Fig. 4D).

### Colchicine-Induced hRGC Axonopathy Disrupts AIS Structure

**Figure 5.**
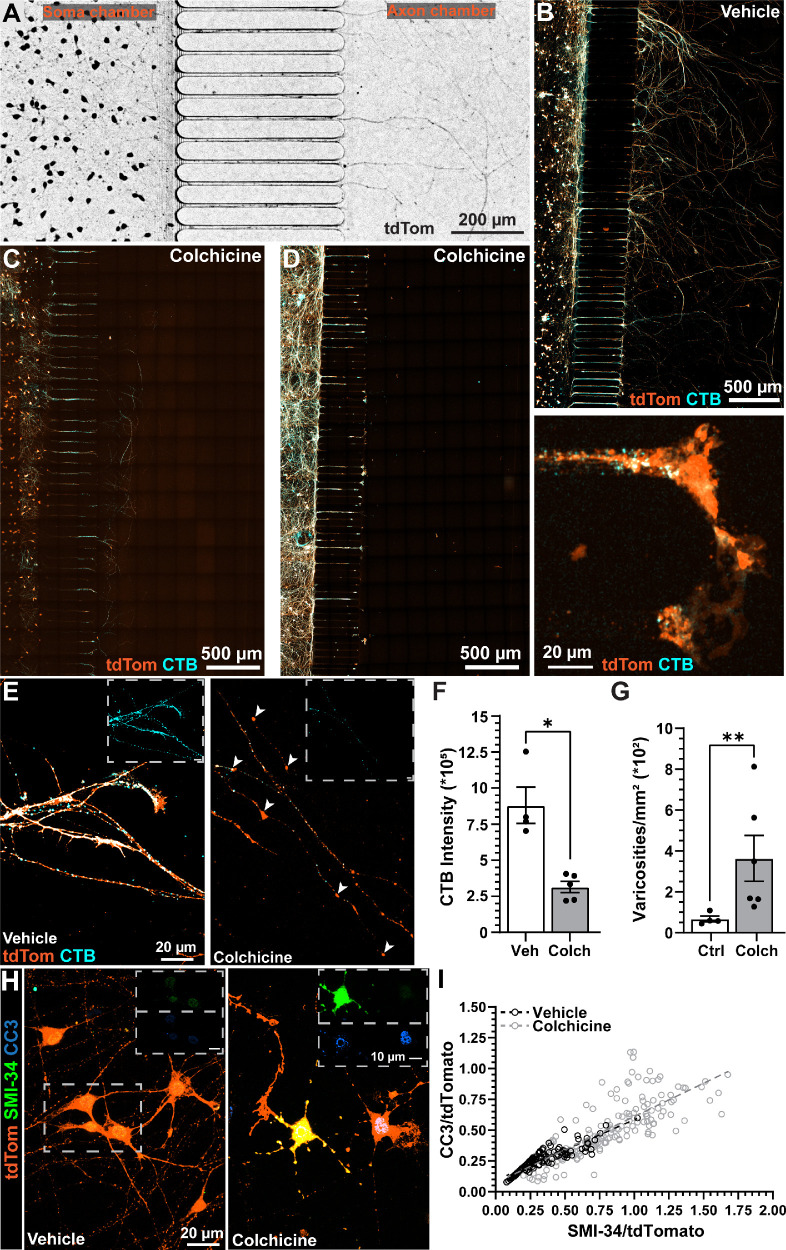
Colchicine application models axonopathy in hRGCs cultured on microfluidic platforms. (**A**) Epifluorescence micrograph of live tdTomato-positive (*black*) hRGCs after 8 DIV, demonstrating extension of putative axons into the axon chamber prior to application of colchicine. (B–D) Representative images of tdTomato-positive hRGCs (*orange*) cultured in microfluidic devices after the addition of either vehicle (**B**) or colchicine (30 nM, 3 days) to the axon chamber (**C, D**). Colchicine caused axon retraction in some samples (**C**) and outright axon degeneration in other samples (**D**). (**D**, *right*) Example degenerative axon located within the microgroove section of the microfluidic platform after colchicine treatment. Fluorescently conjugated CTB (*cyan*) added to the soma chamber (*left side* of images) was transported anterogradely to distal putative axons in the axon chamber. Colchicine appeared to reduce the transport of CTB to spared axons in the axon chamber (**C–E**). (**E**) Higher-magnification example images of the axon chambers in vehicle- and colchicine-treated cultures. CTB fluorescence (*cyan*) is evident in the vehicle samples but was reduced after colchicine treatment. In colchicine-treated cultures, spared axons exhibited degenerative varicosities (**E**, *right*, *white arrowheads*). (**F, G**) After colchicine treatment, CTB fluorescence in the axon chamber was significantly reduced (*P* = 0.0159), and varicosity density significantly increased (*P* = 0.0095). The vehicle group had four independent samples, and the colchicine group had five or six independent samples. (**H**) Example confocal images of hRGCs (*orange*) after vehicle (*left*) or colchicine (*right*) treatment and immunolabeled against SMI-34 (*green*) and CC3 (*blue*). *Scale bars*: 10 µm (**E, H**). (**I**) We found significant positive correlations between CC3 and SMI-34 for both vehicle-treated cells (*R*^2^ = 0.80, *P* < 0.001) and colchicine-treated cells (*R*^2^ = 0.72, *P* < 0.001). CC3 and SMI-34 integrated density was normalized by tdTomato fluorescence integrated density. The vehicle group included 123 cells from two devices, and the colchicine group included 214 cells from four devices. Statistics: Mann–Whitney test (**F, G**), linear regression (**I**). *Error bars*: ±SEM. *P < 0.05, **P < 0.01.

Next, we sought to develop an in vitro model of axonopathy in hRGCs cultured in microfluidic platforms. In live samples, we noted that putative axons extended through the microgroove barrier and into the axon chamber within 8 to 10 DIV (Fig. 5A). Allowing for additional time in vitro for axon elongation, after 10 to 12 DIV we administered either vehicle (culture medium) or 30-nM colchicine into the axon chamber. Two days after the treatment with vehicle or colchicine, we added 1% CTB 647 into the soma chamber of the microfluidic platform to evaluate anterograde axonal transport. Previous investigations on anterograde axonal transport using neural tracers have demonstrated that similar microfluidic platforms maintain fluidic isolation between chambers for at least 20 hours.^26^ Twelve hours later, we prepared samples for immunocytochemistry and confocal microscopy.

In vehicle conditions, we observed tdTomato-positive, axon-like processes labeled with CTB extending into the axon chamber up to 2 mm from the soma chamber (Fig. 5B). Following treatment with colchicine, axons appeared retracted or outright degenerated (Figs. 5C, 5D). In spared axons, we observed enlarged varicosities following colchicine treatment and ostensibly diminished CTB transport relative to vehicle-treated hRGC axons (Fig. 5E). Quantification of CTB fluorescence within remaining fibers in the axon chamber indicated that colchicine treatment significantly reduced anterograde transport by 64% (*P* = 0.0159) (Fig. 5F). In addition to axon transport deficits, we found that colchicine significantly increased the density of axonal varicosities by 438% (*P* < 0.0095) (Fig. 5G). Finally, we probed hRGCs for additional indicators of degeneration, including expression of phosphorylated neurofilament H (SMI-34) and cleaved caspase 3 (CC3) in the somas.^39^^,^^40^ As expected, based on our results from axon transport assays, we found that colchicine treatment appeared to increase the accumulation of both SMI-34 and CC3 within hRGC somas (Fig 5H). We determined the relationship between SMI-34 and CC3 expression and the influence of colchicine treatment by normalizing SMI-34 and CC3 immunofluorescence to tdTomato fluorescence and plotting the resultant values for each cell. Colchicine did not significantly affect tdTomato fluorescence (vehicle, 155,580 ± 49,457 integrated density; colchicine, 83,172 ± 33,006 integrated density; *P* = 0.228). Following normalization, we found significant positive correlations between SMI-34 and CC3 for both vehicle and colchicine conditions (*P* < 0.001). We found 0.80 and 0.72 of the variation in CC3 immunofluorescence accounted for by SMI-34 immunolabeling in vehicle and colchicine samples, respectively. Notably, we observed colchicine increased both SMI-34 and CC3 intensity in many cells compared to vehicle (Fig. 5I).

**Figure 6.**
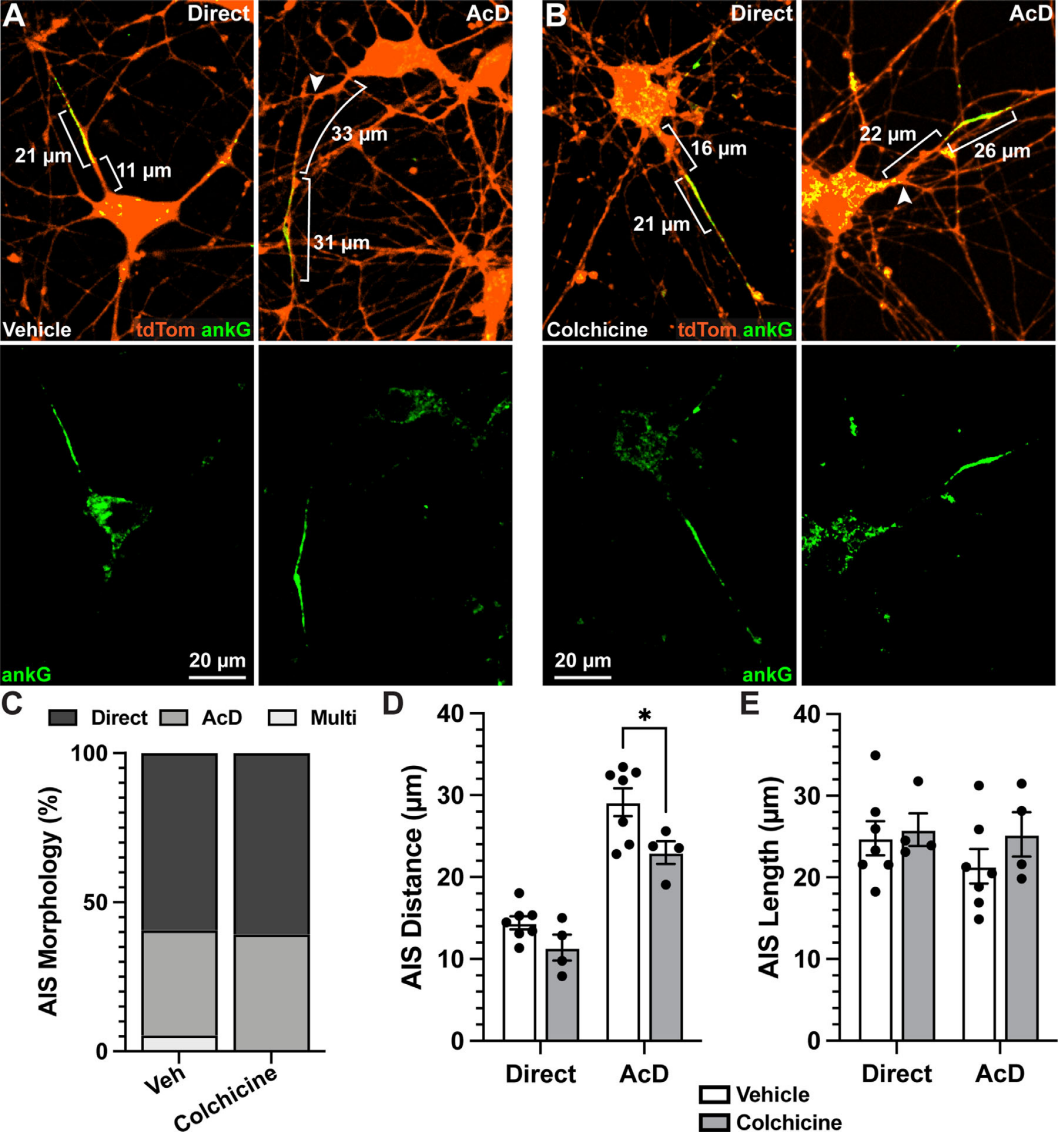
AcD axon initial segments are preferentially susceptible to colchicine-induced changes in morphology. (**A, B**) Example images of ankG labeling (*green*) in hRGCs (*orange*) treated with vehicle (**A**) or colchicine (**B**). (**C**) Cumulative percentages of AIS localizations for both treatment groups. No multi-AIS cells were seen in the colchicine group, although there was not a significant difference in the distribution of localizations between groups (*P* = 0.104). (**D**) Colchicine treatment significantly decreased AIS distance from the soma (*P* = 0.0063), with a greater decrease seen in AcD AISs (*P* = 0.0179) than in direct AISs (*P* = 0.3041). (**E**) Colchicine treatment did not significantly affect AIS length (*P* = 0.3066). The vehicle group included 190 cells from seven independent devices, and the colchicine group included 76 cells from four independent devices. Statistics: χ^2^ test (**C**), two-way ANOVA with Šídák's multiple comparisons test (**D, E**). *Error bars*: ±SEM. *P < 0.05.

Finally, we sought to determine the influence of colchicine on AIS morphology; however, we found that colchicine degraded ankG immunofluorescence, so we could not identify an AIS for many hRGCs. Therefore, we evaluated AIS localization and dimension on cells with visible ankG labeling for which we could trace its somatic origin, and we found instances of ankG enrichment on a process stemming directly from the soma and on AcDs (Figs. 6A, 6B). We did not observe any colchicine-treated hRGCs possessing multiple AISs projecting from a single soma; however, the overall distribution of AIS localizations was not statistically different between vehicle and colchicine-treated cells (*P* = 0.1041) (Fig. 6C). As described above (Fig. 3), we then measured the distance from the soma and length of each AIS. We found the AISs remaining after colchicine treatment tended to be closer to the soma overall than vehicle conditions (*P* = 0.0063) (Fig. 6D). AISs localized to AcDs appeared to be more strongly impacted by colchicine treatment (*P* = 0.0179) (Fig. 6D) than direct AISs (*P* = 0.3041) (Fig. 6C). Although colchicine significantly reduced the distance from the soma of ankG labeling in AcDs, colchicine did not impact AIS length for either AIS type in the remaining cells (*P* = 0.3066) (Fig. 6E).

## Discussion

Previously, we observed that hRGCs cultured on glass coverslips without growth factor supplementation did not form well-defined AISs.^12^ Here, we sought to develop an in vitro model to drive hRGC axon specification. We demonstrated that microfluidic platforms promote polarization based on ankG and PSD-95 localization. Using this microfluidic platform, next we sought to develop and validate a model of axonopathy and test the influence of injury on AIS morphology. We chemically lesioned hRGC axons by adding colchicine, and we found that AcD AISs respond to insult through changes in morphology.

We demonstrated a foundation for characterizing hRGC AIS morphology by first examining mouse RGC AIS morphology. Our characterization of mouse RGCs AIS morphologies revealed direct and AcD profiles similar to those previously observed in hippocampal pyramidal, cortical, and RGCs (Figs. 1A, 1B).^27^^,^^28^^,^^36^^,^^41^^–^^43^ Moreover, our AIS geometry measurements of mouse RGCs were consistent with previously reported values.^28^^,^^43^^–^^45^

Compared to mouse RGCs, hRGCs plated on coverslips exhibited heterogeneity in AIS localization and morphology as evidenced by AIS type and dimension variability (Figs. 1C–1H; Figs. 4A, 4D). Importantly, one-fourth of these cells contained multiple ankG-positive putative axons originating from a single soma (Fig. 1G; Fig. 4A). This multi-AIS phenotype can be induced by systemic pharmacologic enhancement of microtubule stability or disrupting the binding of ankG to the cytoskeleton,^46^^,^^47^ suggesting that multi-AIS cells lack axon specification. Culturing hRGCs on microfluidic platforms reduced the number of multi-AIS cells and increased the proportion of cells with direct AISs, indicating that microfluidic platforms promote axon specification and normalized morphology relative to mouse RGCs (Fig. 2C, Figs. 4A–4D).

Based solely on this set of experiments, the mechanisms underlying the increase in the number of polarized neurons in microfluidic devices are unclear. However, evidence suggests that axon specification may be enhanced by promoting long-range neurite outgrowth through the restricted confines of the microgroove section of microfluidic devices, which inhibit neurites from turning back toward the soma chamber and growth factor gradients generated through hydrostatic pressure.^26^^,^^48^ The initial group of cells containing pioneering axons may increase polarization of neighboring neurons.^49^ Future studies will test this notion directly by comparing AIS localization and morphology in cells extending long-ranged axons versus cells that do not.

AIS geometry is an indicator of neuronal development, synaptic strength, and intrinsic excitability.^36^^,^^44^^,^^50^^–^^56^ Considering results from others, the AIS geometry of hRGCs, regardless of culture platform, predicts hyperexcitability compared to the AIS geometry of mouse RGCs.^29^^,^^57^ Our earlier findings support this estimate. We found hRGCs that were sensitive to small depolarizing currents (10 pA), but hRGCs produced depolarization block when the depolarizing current strength was modestly increased (20–60 pA).^12^ This small dynamic range for repetitive firing may be due to weak synaptic input, reduced expression of voltage-sensitive channels, or improper localization of voltage-gated channels along the AIS.^53^^,^^58^ Regarding synapses, evidence suggests that coculturing human stem cell–derived RGCs with astrocytes increases localization of postsynaptic densities and repetitive firing; yet, cells remain sensitive to depolarization block.^22^ As depolarization block is primarily due to an accumulation of inactivation of voltage-gated Na^+^ (Na_V_) channels,^59^ future investigations will probe the influence of culture platform on expression and localization of Na_V_1.2 and Na_V_1.6 channels relative to ankG in hRGCs.

Next, we sought to develop and validate an in vitro model of hRGC axonopathy by chemically lesioning axons using colchicine and testing the influence of injury on AIS morphology. Here, we found that colchicine produced remarkable axon retraction and degeneration (Figs. 5C, 5D). This variability in the responses of axons to colchicine between cultures may be due to modest volume differences between the soma and axon chambers that led to dilution of the colchicine in the axon chamber. If this is true, then the application of membranes covering the access ports of the microfluidic platforms may prevent evaporation that might produce slight differences in volume between chambers. In samples that contained neurites in the axon chamber, we found that colchicine reduced anterograde axonal transport of CTB and increased the density of enlarged varicosities (Figs. 5E–5G). In agreement with these results, several studies have shown that axonopathy produces deficits in anterograde axonal transport and induces the formation of varicosities.^60^^–^^64^

Deficits in axonal transport and varicosity growth are two interrelated markers of degeneration, as axonal transport of mitochondria ceases during varicosity formation.^64^ Interestingly, evidence from both in vitro and in vivo preparations suggests that varicosity development is partially reversible, indicating that the underlying mechanisms of varicosity formation may be a target for neuronal repair.^64^^–^^66^ Based on our findings from axonal transport and morphologic assays, we were not surprised to find that colchicine increased expression of other indicators of degeneration, including SMI-34 and CC3 (Figs. 5H, 5I).^39^^,^^40^ Overall, our results suggest that microfluidic platforms provide a format to model axonopathy and test mechanisms for neuronal repair.

Finally, we examined if colchicine-induced axonopathy altered AIS morphology in hRGCs (Figs. 6A, 6B). Interestingly, hRGC AcDs appeared to be more sensitive to colchicine than direct AISs. Following colchicine treatment, AcDs produced a proximal shift toward the soma (Fig. 6D). This finding suggests at least three possibilities: Some hRGCs are less vulnerable to injury, cells with AcDs closer to the soma are less susceptible to stress, or cells containing AcDs respond to stress through changes in AIS geometry. The mouse retina contains over 40 different types of RGCs, and some of them appear to be less sensitive to stress.^43^^,^^67^^–^^71^ Of note, RGCs that possess large somas expressing modest amounts of melanopsin and produce a sustained response to light onset (i.e., αON-sustained/M4 RGCs) seem to be less vulnerable to injury.^67^^,^^68^^,^^72^^,^^73^ However, in a cell line similar to the one used in these studies, melanopsin-positive cells are sparse.^74^ Alternatively, several studies have indicated that AIS geometry is plastic, changing in an activity-dependent manner^50^^,^^51^^,^^57^^,^^75^ and becoming altered in degenerative disease.^31^^,^^32^ Future studies will test local stabilization of ankG-associated microtubules during axonopathy toward protecting axons during stress.^20^^,^^47^
